# Butanol Dehydration over V_2_O_5_-TiO_2_/MCM-41 Catalysts Prepared via Liquid Phase Atomic Layer Deposition

**DOI:** 10.3390/ma6051718

**Published:** 2013-04-29

**Authors:** Hyeonhee Choi, Jung-Hyun Bae, Do Heui Kim, Young-Kwon Park, Jong-Ki Jeon

**Affiliations:** 1 Department of Chemical Engineering, Kongju National University, Cheonan 331-717, Korea; E-Mails: chh9010@kongju.ac.kr (H.C.); junghyun@kongju.ac.kr (J.-H.B.); 2 School of Chemical and Biological Engineering, Institute of Chemical Processes, Seoul National University, Seoul 151-742, Korea; E-Mail: dohkim@snu.ac.kr; 3 School of Environmental Engineering, University of Seoul, Seoul 130-743, Korea; E-Mail: catalica@uos.ac.kr

**Keywords:** butanol, dehydration, MCM-41, atomic layer deposition, 1-butene, vanadium oxide, titanium oxide

## Abstract

MCM-41 was used as a support and, by using atomic layer deposition (ALD) in the liquid phase, a catalyst was prepared by consecutively loading titanium oxide and vanadium oxide to the support. This research analyzes the effect of the loading amount of vanadium oxide on the acidic characteristics and catalytic performance in the dehydration of butanol. The physical and chemical characteristics of the TiO_2_-V_2_O_5_/MCM-41 catalysts were analyzed using XRF, BET, NH_3_-TPD, XRD, Py-IR, and XPS. The dehydration reaction of butanol was performed in a fixed bed reactor. For the samples with vanadium oxide loaded to TiO_2_/MCM-41 sample using the liquid phase ALD method, it was possible to increase the loading amount until the amount of vanadium oxide reached 12.1 wt %. It was confirmed that the structural properties of the mesoporous silica were retained well after titanium oxide and vanadium loading. The NH_3_-TPD and Py-IR results indicated that weak acid sites were produced over the TiO_2_/MCM-41 samples, which is attributed to the generation of Lewis acid sites. The highest activity of the V_2_O_5_(12.1)-TiO_2_/MCM-41 catalyst in 2-butanol dehydration is ascribed to it having the highest number of Lewis acid sites, as well as the highest vanadium dispersion.

## 1. Introduction

Metal oxides are important catalysts in the petrochemical and fine chemical industries. In the catalytic reaction, supported metal oxides are used for dehydration, ethylene polymerization, isomerization, selective catalytic reduction of nitrogen oxides, and so forth. Among the catalysts, vanadium oxide is attracting much research interest, because of its many possible uses in a variety of chemical processes, due to its unique characteristics and high catalyst efficiency. Many studies dealing with the characteristics of vanadium oxide supported on oxides have been published [1,2,3,4]. Silica is known to be inadequate as a support for vanadium oxide, due to the weak interaction between vanadium oxide and the silica surface. Among all oxides, only zirconium oxide and titanium oxide appear to be viable options to achieve high dispersion through strong interaction with VO*_x_*. However, zirconium oxide and titanium oxide do not have enough surface area to achieve a high loading amount and a high level of dispersion simultaneously.

Therefore, to achieve the goal of obtaining highly loaded and dispersed vanadium oxide, an appropriate support is essential. Furthermore, to date there have been few reports on the use of mesoporous silica as a support for vanadium oxide. Most studies suggest that since the Si–O–V bonds are at least partially broken during the heat treatment, the vanadium oxide being supported is very unstable, and that this low stability is a major obstacle to the use of mesoporous materials to support vanadium oxide. From this perspective, to support vanadium oxide it is necessary to devise a way to use a mesoporous material with a monolayer of titanium oxide attached to its surface.

Wang* et al.* reported a method to produce a highly dispersed metal oxide on the surface of mesoporous silica using the atomic layer deposition (ALD) method in the liquid phase [5]. This method, devised by Ichinose* et al.*, is a surface sol-gel process, and was originally employed to produce a thin metal oxide film on a two dimensional (2D) surface [6]. The Ichinose method is a 4-step process. The first step involves chemical adsorption of alkoxide in the liquid phase; the second step involves washing the remaining alkoxide precursor that has not been adsorbed with a solvent; the third step is hydrolysis of the chemically adsorbed alkoxide; and the last step involves a drying process. This method can be applied to form different monolayers of metal oxides, including zirconium oxide, vanadium oxide, and titanium oxide, on the surface of mesoporous silica.

Recently, there has been an imbalance between demand and supply in the butenes market, due to rising naphtha prices according to rising oil prices. In addition, the ethane cracking process has been newly established, and is expanding at an accelerating pace. Since the ethane cracking process produces a large amount of ethylene but no C_4 _derivatives, there is an overall shortage of C_4 _derivatives. The supply shortage will worsen and the prices will continue to rise unless a new raw material is developed. In response, many researchers and companies are currently attempting to use biomass—a sustainable fuel—to produce hydrocarbons, as well as other techniques to produce butenes from bio-butanol [7,8,9,10,11,12]. 

In this research, MCM-41—a mesoporous silica—was used as a support. By using atomic layer deposition (ALD) in the liquid phase, a catalyst was produced by consecutively loading titanium oxide and vanadium oxide to the support. This study focused on analyzing the effects of the loading amount of vanadium oxide on the acidic characteristics and the effectiveness of the dehydration reaction of butanol. The physical and chemical characteristics of a TiO_2_-V_2_O_5_/MCM-41 catalyst were analyzed using XRF, BET, NH_3_-TPD, XRD, Py-IR, XPS, and so forth. The dehydration reaction of butanol was performed in a fixed bed reactor.

## 2. Experimental Details

MCM-41 was prepared following procedures described in the literature [13,14]. Titanium oxide was added on MCM-41 using the ALD method [8]. MCM-41 was suspended in anhydrous toluene and refluxed at 100 °C for 3 h under bubbled N_2_. The titanium precursor (titanium (IV) isopropoxide) was suspended in anhydrous toluene and refluxed for 6 h under bubbled N_2_. The solution including MCM-41 was then mixed with the precursor solution and refluxed for 15 h under bubbled N_2_. After washing with toluene, the mixed solution was filtered, dried in a 120 °C oven for 30 min, and finally calcined at 500 °C. The resultant solid was refluxed again in toluene and then vanadium triethoxide was added to the toluene suspension. The amount of added vanadium triethoxide corresponds to that needed to obtain a loading of 2.6–12.1 wt % vanadium oxide. The mixture was refluxed, filtered, dried, and calcined at 500 °C. This sample is referred to as V_2_O_5_-TiO_2_/MCM-41. 

Inductively coupled plasma-atomic emission spectroscopy (ICP-AES; Flame Modula S, Spectro, Germany) was used to analyze for titanium and vanadium in the sample. Before these analyses, a sample pre-processor (Milstone/Ethos Touch Control) was used, where 7 mL of nitric acid and 2 mL of hydrochloric acid were added to 0.2 g of the sample, followed by heating at 453 K for 17 min before sample introduction to the ICP-AES instrument. The crystallinity of the catalysts was investigated using an X-ray diffractometer (XRD). XRD was obtained from a Rigaku D/MAX-II device using Cu Kα radiation energy, and small-angle powder XRD patterns were recorded on a Rigaku D/max-2500 X-ray diffractometer. Nitrogen adsorption–desorption isotherms were determined using a Micromeritics ASAP 2020. The surface area was calculated according to the BET equation. The pore volume was obtained by the *t*-plot method. X-ray photoelectron spectroscopy (XPS) was used to analyze the oxidation state of vanadium oxide on the samples [8]. The XPS analyses were conducted on a MultiLab ESCA 2000 X-ray photoelectron spectrometer with MgKa radiation at 300 W. 

The acidic property of the samples was analyzed using temperature-programmed desorption (TPD) of chemisorbed ammonia [15,16]. The natures of the acid sites were investigated using pyridine as the probe molecule [13,15]. Pyridine vapor was admitted in doses until the surface of the catalyst wafer was saturated. Infra-red spectra over a wafer that contained chemisorbed pyridine were recorded using a Spectrum GX (Perkinelmer) with an MCT detector at a temperature range of 100–350 °C. 

2-Butanol dehydration reaction was performed using a fixed bed reactor containing 0.01 g of the catalyst. After maintaining the reactor temperature at a fixed level under a nitrogen flow of 200 mL/min, 2-butanol was supplied in a flow of 1 ml/hr. In this case, the WHSV was 80 h^−1 ^(for butanol). A syringe pump was used to inject 2-butanol into the reactor at a fixed rate. The N_2_ gas flow was regulated with a mass flow controller, and the reactor temperature was controlled with a tubular furnace equipped with a programmable temperature controller. The gas phase products were analyzed using an online gas chromatograph (YL 6100 GC) equipped with FID and an alumina capillary column.

## 3. Results and Discussion 

### 3.1. Catalyst Characterization

The XRF analysis results of the loading amount of titanium oxide and vanadium oxide in samples that were produced using the atomic layer deposition method under a liquid phase are indicated in Table 1. It is known that the stability of vanadium oxide is low when mesoporous silica is used as the support. Herrera *et al.* reported a method to load vanadium oxide onto the surface of mesoporous silica with an attached monolayer of titanium oxide by using atomic layer deposition [9,17]. In the research presented here, the loading amount of titanium oxide in a sample that had only loaded titanium oxide (TiO_2_/MCM-41) using the liquid phase ALD method was 8.4 wt %. For the samples with vanadium oxide loaded to a TiO_2_/MCM-41 sample using the liquid phase ALD method, it was possible to increase the loading amount until the amount of vanadium oxide reached 12.1 wt %. 

**Table 1:** Loading amount of metal oxides on MCM-41 determined by inductively coupled plasma (ICP).

Catalyst	TiO_2_ (wt %)	V_2_O_5_ (wt %)
TiO_2_/MCM-41	8.4	–
V_2_O_5_(2.6)-TiO_2_/MCM-41	7.6	2.6
V_2_O_5_(3.6)-TiO_2_/MCM-41	7.5	3.6
V_2_O_5_(7.6)-TiO_2_/MCM-41	7.0	7.6
V_2_O_5_(12.1)-TiO_2_/MCM-41	7.1	12.1

**Figure 1:**
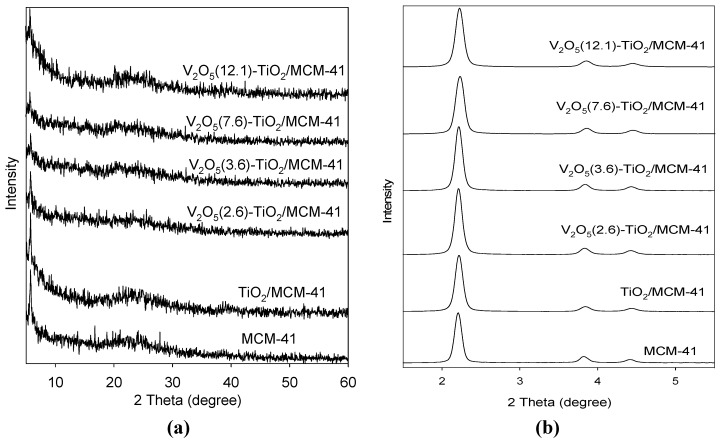
XRD patterns of various catalysts (**a**) low angle XRD; (**b**) high angle XRD.

Figure 1 shows the XRD patterns of V_2_O_5_-TiO_2_/MCM-41catalysts. As shown in the low angle XRD patterns, all of the samples exhibited an intense peak and two small peaks, corresponding to peaks at (100), (110), and (200), which are characteristic of a 2-dimensional hexagonal mesostructure [7,14]. The high-angle XRD patterns of TiO_2_/MCM-41 and V_2_O_5_-TiO_2_/MCM-41catalysts show no diffraction intensity, except for the peak that corresponds to amorphous silica, thereby implying that vanadium oxides, as well as titanium oxides, are highly dispersed on the support materials. 

**Figure 2:**
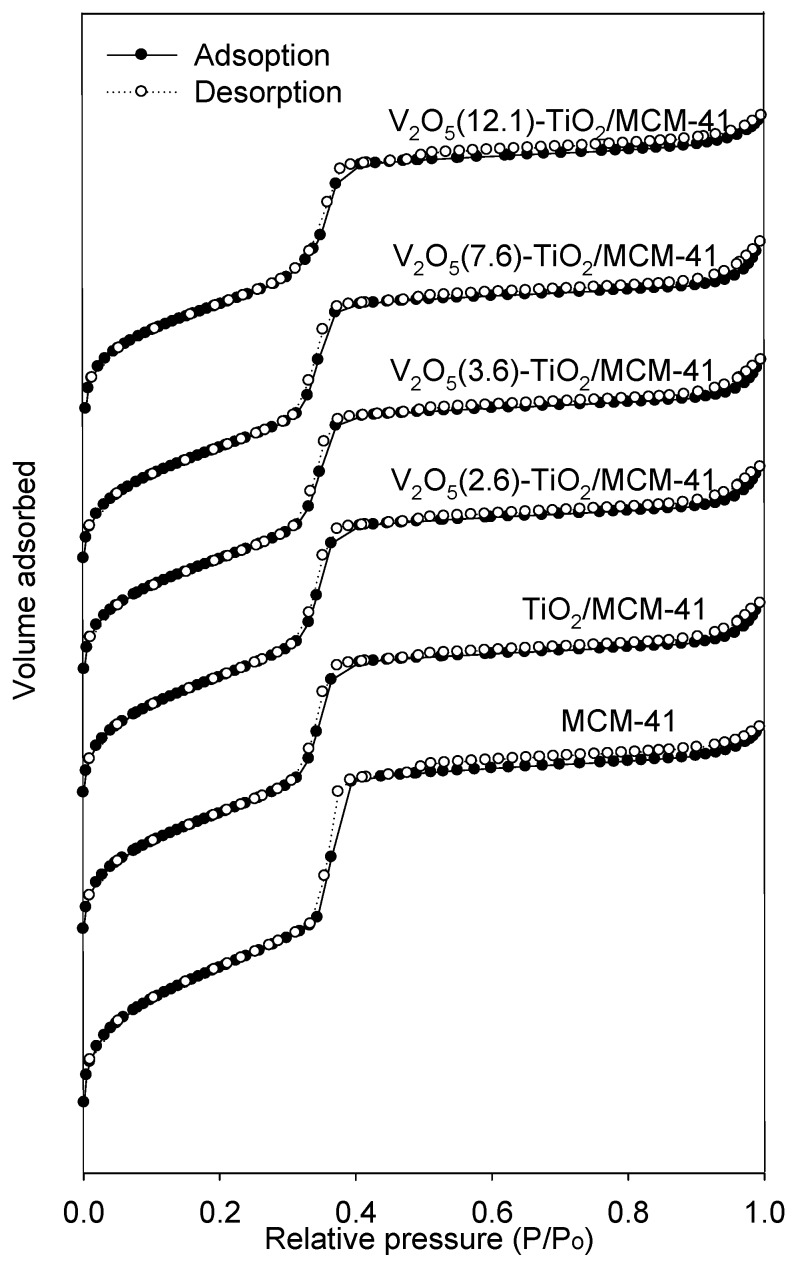
N_2_ adsorption–desorption isotherms of various catalysts.

N_2_ adsorption–desorption isotherms of the MCM-41, TiO_2_/MCM-41, and V_2_O_5_-TiO_2_/MCM-41 samples are shown in Figure 2. All of these materials exhibit a Type IV isotherm, which, according to the IUPAC nomenclature, is characteristic of a mesoporous material [18]. Textural parameters of all the catalysts are summarized in Table 2. After introducing the titanium precursor, the BET surface area and pore volume decreased slightly. This may be due to partial pore blockage by the introduction of titanium oxide. Furthermore, the surface area and pore volume of the V_2_O_5_-TiO_2_/MCM-41 catalysts decreased with increasing vanadium oxide loading up to 12.1 wt %. As illustrated by XRD, vanadium oxide and titanium oxide might form highly dispersed, small particles. Therefore, the particles can be located mainly in the mesopores and are well distributed over the internal surface. This might result in a decrease in the surface area of V_2_O_5_-TiO_2_/MCM-41. 

**Table 2:** BET surface area and pore volume of the catalysts.

Catalyst	S_BET_ (m^2^/g)	V_p_ (cm^3^/g)
MCM-41	1069	0.99
TiO_2_/MCM-41	955	0.84
V_2_O_5_(2.6)-TiO_2_/MCM-41	954	0.87
V_2_O_5_(3.6)-TiO_2_/MCM-41	898	0.82
V_2_O_5_(7.6)-TiO_2_/MCM-41	907	0.84
V_2_O_5_(12.1)-TiO_2_/MCM-41	859	0.78

**Figure 3:**
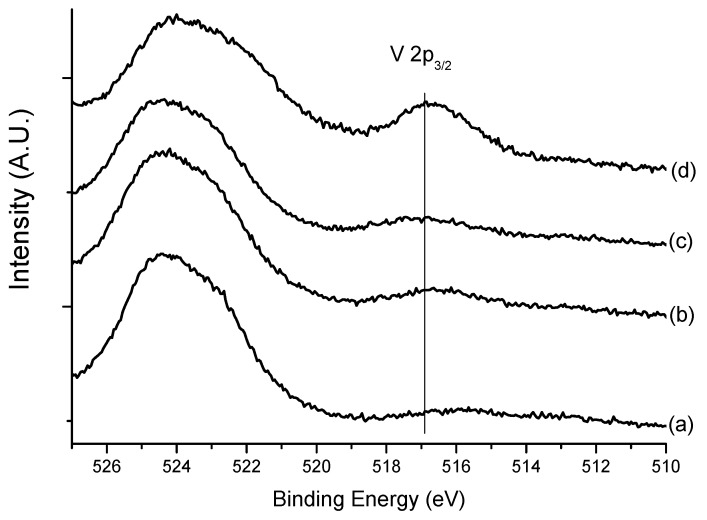
V 2p XPS of various samples: (**a**) V_2_O_5_(2.6)-TiO_2_/MCM-41; (**b**) V_2_O_5_(3.6)-TiO_2_/MCM-41; (**c**) V_2_O_5_(7.6)-TiO_2_/MCM-4; and (**d**) V_2_O_5_(12.1)-TiO_2_/MCM-41.

In order to investigate dispersion of vanadium species on the sample, X-ray photoelectron spectroscopy in the vanadium 2p region was applied to various samples with different vanadium loading. Figure 3 shows the peak around 525 eV originating from the oxygen satellite peak, when a Mg Kα X-ray source was used. A small peak at 517 eV is assigned to V(5+) species in the sample [19], as marked in the line of the figure. With an increasing amount of vanadium—from 2.6 wt % to 7.6 wt %—the peak area of V 2p_3/2 _increases slightly. Moreover, when the vanadium loading reaches 12.1 wt %, the peak intensity of V 2p_3/2 _is substantially enhanced, implying that in this range the additional vanadium species becomes well dispersed on the surface. These high vanadium dispersion results obtained from XPS correspond well to the activity results—that the sample with 12.1 wt % vanadium loading has maximum activity. In other words, the close relationship between vanadium dispersion and the activity is elucidated.

**Figure 4:**
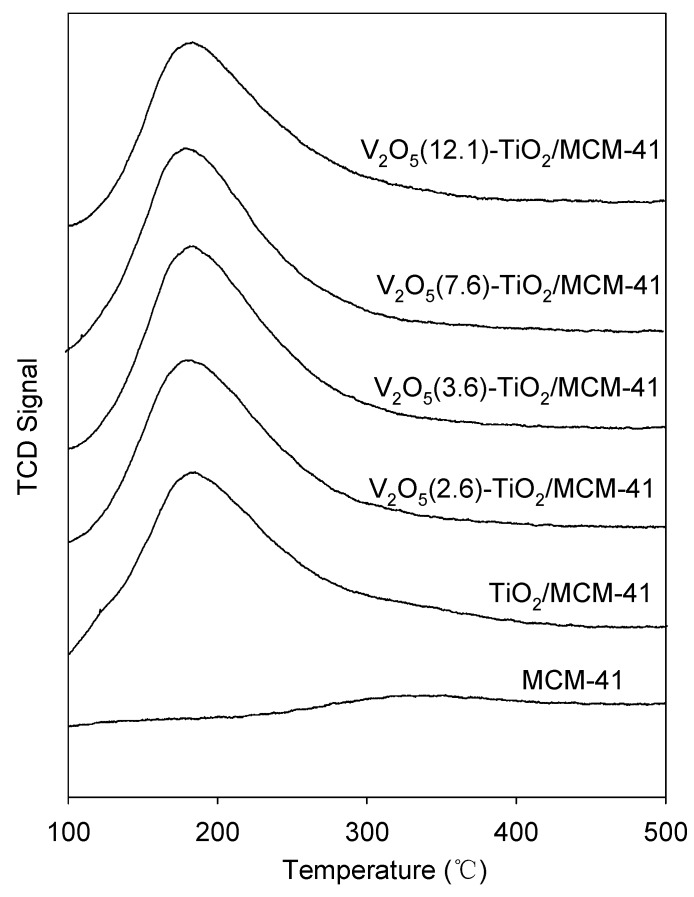
Temperature-programmed desorption of ammonia over various catalysts.

Figure 4 shows the changes in the NH_3_-TPD profiles with titanium oxide and vanadium oxide loading in MCM-41. As expected, the MCM-41 sample did not show any peak in NH_3_-TPD, which indicates that there are few acid sites on MCM-41. The TiO_2_/MCM-41 samples showed weak acid sites only at around 180 °C, whereas strong acid sites were not observed, thus showing that only weak acid sites were produced upon titanium oxide loading. However, with further loading of vanadium oxide up to 12.1 wt %, the number of acid sites, calculated from the peak area of NH_3_ desorption, did not increase with increasing vanadium oxide loading. 

**Figure 5:**
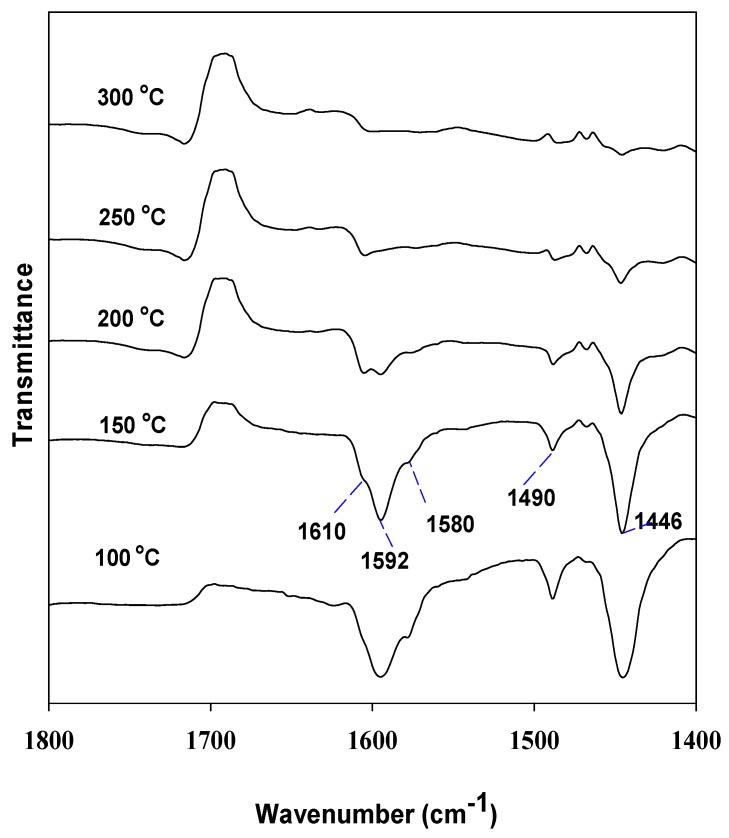
FT-IR of adsorbed pyridine over V_2_O_5_(12.1)-TiO_2_/MCM-41 sample. The sample was desorbed under 10^−3^ torr at 100, 150, 200, 250, and 300 °C.

Figure 5 shows the FT-IR spectra of the pyridine adsorbed on the V_2_O_5_(12.1)-TiO_2_/MCM-41 sample, followed by the desorption at elevated temperatures from 100 to 300 °C. At 100 °C, two bands at 1592 cm^−1 ^and 1446 cm^−1 ^were dominant among the bands. An increase in the desorption temperature seems to reduce the intensity of the band at 1592 cm^−1 ^and 1446 cm^−1 ^more drastically than that of the bands at 1610 cm^−1^, 1580 cm^−1^, and 1490 cm^−1^. It is clear that the bands at 1610 cm ^−1^, 1580 cm^−1^, and 1490 cm^−1^ show the presence of a higher strength acid site than do the bands at 1592 cm^−1 ^and 1446 cm^−1^. These trends are consistent with those reported in the literature, and three bands at 1610 cm^−1^, 1580 cm^−1^, and 1490 cm^−1^ could be assigned to Lewis acid sites, while the bands at 1592 cm^−1 ^and 1446 cm^−1^ to weak acid sites (H-bonded to surface OH groups) [20,21]. 

**Figure 6:**
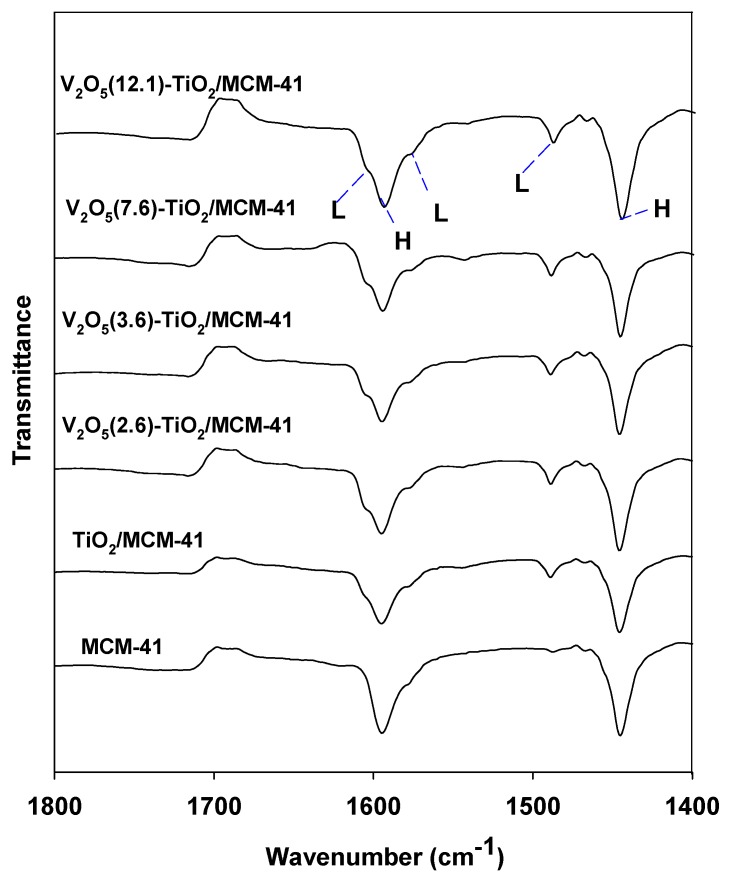
FT-IR of adsorbed pyridine over various samples (150 °C, 10^−3^ torr).

Figure 6 shows the infra-red spectra of the pyridine adsorbed on the samples. The MCM-41 sample does not have Brönsted (B) acid sites or Lewis (L) acid sites, but H sites generated by hydrogen-bonded pyridine (H). Lewis acid sites were produced on the TiO_2_/MCM-41 samples, and are attributed to the creation of titanium-oxide-induced acid sites. It is noticeable that the V_2_O_5_(12.1)-TiO_2_/MCM-41 sample shows the highest intensity of the bands at 1490, 1580, and 1610 cm^−1^, which demonstrates that the V_2_O_5_(12.1)-TiO_2_/MCM-41 catalyst has the greatest amount of Lewis acid sites among the catalysts.

### 3.2. 2-Butanol Dehydration

**Figure 7:**
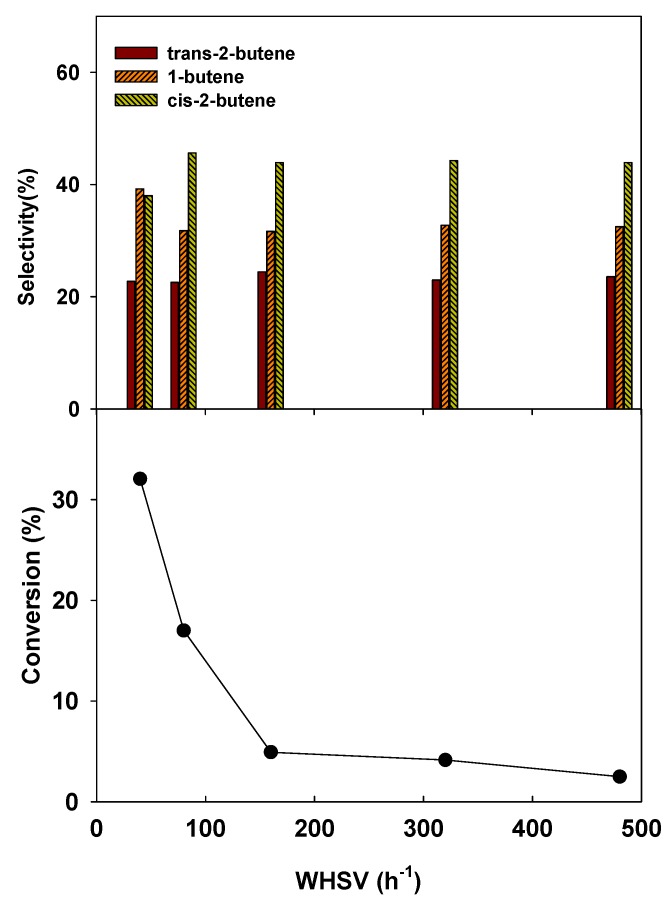
Effect of space velocity on conversion and selectivity in 2-butanol dehydration (Reaction condition: 250 °C, 1 atm, catalyst: V_2_O_5_(12.1)-TiO_2_/MCM-41).

The effect of space velocity on 2-butanol dehydration over V_2_O_5_(12.1)-TiO_2_/MCM-41 catalyst was investigated (Figure 7). When the WHSV was increased from 40 to 160 h^−1^, 2-butanol conversion decreased drastically. A slight decrease in 2-butanol conversion was observed, however, when the WHSV was increased from 160 to 480 h^−1^. When the WHSV was increased from 40 to 80 h^−1^, the selectivity to 1-butene decreased, while that of *cis*-2-butene increased. In the case of a further increase of the WHSV to 480 h^−1^ the product distribution was almost unchanged. Because this observation suggests that there was a kinetically controlled reaction at a higher WHSV, the catalytic performance of the catalysts in this study was investigated at a WHSV of 80 h^−1^ thereafter. In addition, it was noted that 2-butanol hardly reacted without the catalyst under the reaction conditions of 250 °C and 1 atm.

**Figure 8:**
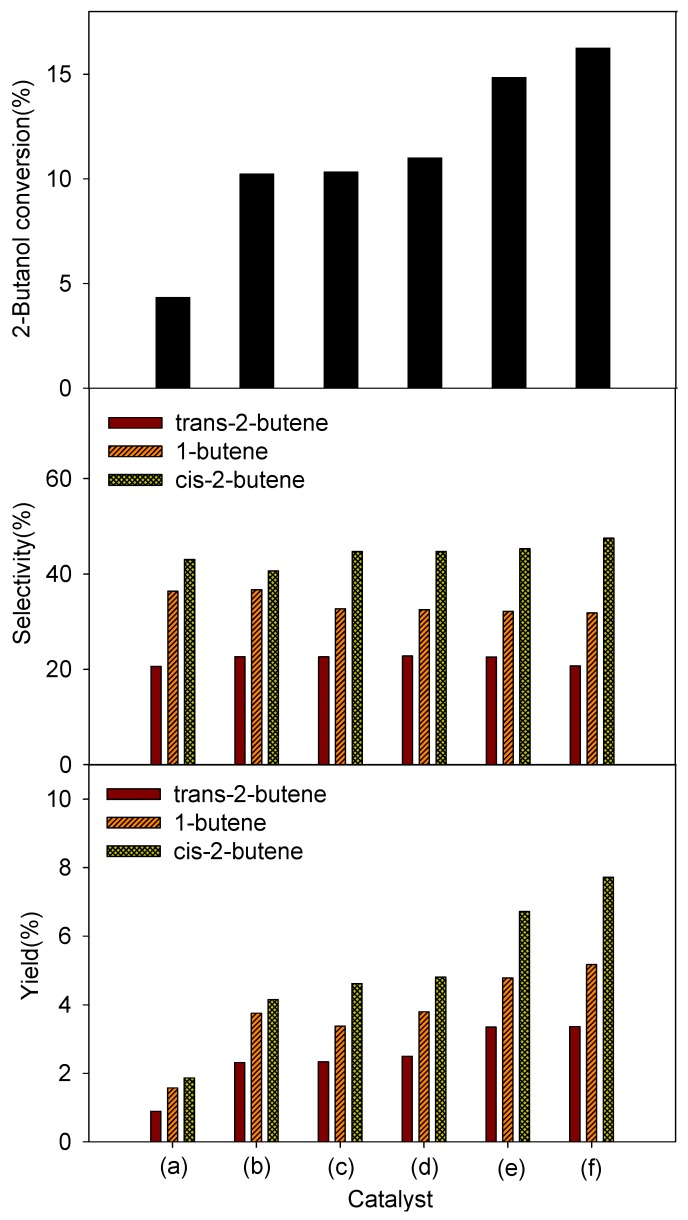
Conversion, selectivity, and yield of 2-butanol dehydration over various catalysts: (**a**) MCM-41; (**b**) TiO_2_/MCM-41; (**c**) V_2_O_5_(2.6)-TiO_2_/MCM-41; (**d**) V_2_O_5_(3.6)-TiO_2_/MCM-41; (**e**) V_2_O_5_(7.6)-TiO_2_/MCM-4; and (**f**) V_2_O_5_(12.1)-TiO_2_/MCM-41. (Reaction condition: 250 °C, 1 atm, WHSV 80 h^−^^1^).

Figure 8 shows the 2-butanol conversion and product distribution. While the pure MCM-41 recorded lower than 5.0% 2-butanol conversion at 250 °C, the 2-butanol conversion significantly increased with titanium oxide loading. This observation was attributed to an increase of the overall number of acid sites by titanium oxide loading, which could be confirmed by NH_3_-TPD results (Figure 4). When the vanadium oxide loading was increased to 12.1%, the 2-butanol conversion increased. The product distribution did not change significantly with increased vanadium oxide loading. Because the total acid amount of the V_2_O_5_-TiO_2_/MCM-41 catalysts did not increase with increasing vanadium oxide loading, as shown in Figure 4, the highest activity over the V_2_O_5_(12.1)-TiO_2_/MCM-41 catalyst could not be explained by the total acid amount of the catalysts. We therefore investigated the characteristics of surface acid sites over the catalysts. It was apparent that the Lewis acid sites were dominant over the V_2_O_5_(12.1)-TiO_2_/MCM-41 among the catalysts, which could be confirmed by IR spectra of pyridine adsorption. Therefore, the highest activity of the V_2_O_5_(12.1)-TiO_2_/MCM-41 catalyst is ascribed to it having the highest number of Lewis acid sites as well as the highest vanadium dispersion. 

## 4. Conclusions 

For the samples with vanadium oxide loaded onto the TiO_2_/MCM-41 sample using the liquid phase ALD method, it was possible to increase the loading amount until the amount of vanadium oxide reached 12.1 wt %. It was confirmed that the structural properties of the mesoporous silica were retained well after titanium oxide and vanadium loading. The NH_3_-TPD and Py-IR results indicated that weak acid sites were produced over the Ti/MCM-41 samples, which is attributed to the generation of Lewis acid sites. The highest activity of the V_2_O_5_(12.1)-TiO_2_/MCM-41 catalyst in 2-butanol dehydration is ascribed to it having the highest number of Lewis acid sites as well as the highest vanadium dispersion.
